# Perioperative nursing care for a parturient with hypertriglyceridemic acute pancreatitis undergoing cesarean section: A case report

**DOI:** 10.1097/MD.0000000000046944

**Published:** 2026-01-09

**Authors:** Yinhao Yang, Juanjuan Lin, Bolun Zhang, Limin Cui, Zhenzhen Yuan

**Affiliations:** a Department of Intensive Care Unit, Xiangyang No. 1 People’s Hospital, Hubei University of Medicine, Xiangyang, China; b Department of Nursing, Xiangyang No. 1 People’s Hospital, Hubei University of Medicine, Xiangyang, China.

**Keywords:** cesarean section, hypertriglyceridemic acute pancreatitis, parturient, perioperative nursing

## Abstract

**Rationale:**

Diagnosing hypertriglyceridemic acute pancreatitis (HTG-AP) in a parturient is challenging due to pregnancy-related physiological changes, and its treatment is fraught with complexities. This single-patient case report serves as a valuable resource for nursing practice of a parturient with HTG-AP and contributes to the formulation of relevant guidelines for this condition.

**Patient concerns:**

The patient was a 32-year-old woman with a history of 5 pregnancies and 1 live birth, and her last menstrual period was on November 13, 2024. At 29 weeks and 4 days of pregnancy, she was transferred from the obstetrics department to the intensive care unit (ICU) due to HTG-AP for treatment.

**Diagnoses:**

The patient was diagnosed with HTG-AP.

**Interventions:**

With maternal and infant safety as the core, a multidisciplinary team collaborates to balance the timing of cesarean section and the treatment of pancreatitis, simultaneously monitoring fetal distress, uterine contractions, and maternal metabolism, and precisely implementing plasma exchange and insulin therapy. Strengthen perioperative airway management to prevent acute respiratory distress syndrome and atelectasis, and provide full-course psychological support and health guidance.

**Outcomes:**

After 5 days of treatment in the ICU, the patient was transferred to the operating room for a cesarean section. The operation was successful and a baby girl was delivered. The newborn was transferred to the pediatric department for further treatment. The mother was then returned to the ICU for continued treatment after the operation. On the fourth day after delivery, the patient’s vital signs were stable and she was transferred back to the obstetrics department for further treatment.

**Lessons:**

Multidisciplinary joint care plays a crucial role in the treatment of pregnancy complicated with HTG-AP. In the future, it is necessary to further explore the standardized nursing pathway for HTG-AP during pregnancy and strategies for improving long-term maternal and infant outcomes.

## 1. Introduction

Hypertriglyceridemic acute pancreatitis (HTG-AP) is a type of acute pancreatitis induced by significantly elevated serum triglyceride levels due to abnormal lipoprotein metabolism. HTG-AP has an insidious cause and is prone to misdiagnosis. Moreover, as its etiology differs from that of biliary acute pancreatitis and alcoholic acute pancreatitis, its targeted diagnostic and therapeutic approaches are distinct from those of other types of acute pancreatitis.^[1]^

While acute pancreatitis in pregnancy is uncommon, HTG-AP constitutes a particularly unique and high-risk subset, which is more common in the middle and late stages of pregnancy and tends to recur frequently. It poses a serious threat to both the mother and the fetus.^[2]^ During pregnancy, the enlarged uterus alters the anatomical positions of abdominal organs, and contractions caused by inflammation and exudate often mask the symptoms of pancreatitis. Due to the atypical nature of the location and characteristics of abdominal pain, early diagnosis of HTG-AP can be quite challenging.^[3,4]^ At the same time, the treatment of HTG-AP faces many contradictions, such as anticoagulation therapy increasing the risk of postpartum hemorrhage, drugs for maintaining pregnancy and promoting fetal lung maturity conflicting with the treatment of acute pancreatitis, and antibiotic selection needs to take into account the safety of the fetus, etc.^[5,6]^ These factors make the nursing of HTG-AP during pregnancy face unique challenges, and it is necessary to precisely grasp the timing and intensity of treatment.

In recent years, with the increase in the proportion of elderly pregnant women and changes in dietary habits, the incidence of HTG-AP during pregnancy has been on the rise.^[7]^ However, most existing guidelines on pancreatitis care are not tailored to the parturient, and reports detailing the nuanced nursing care throughout the cesarean section perioperative period are scarce. This case’s uniqueness lies in its demonstration of a successful, standardized nursing protocol adapted to these dual pressures. It contributes to clinical practice by delineating essential monitoring parameters, emphasizing the timing and coordination of multidisciplinary interventions, and outlining a dynamic care plan that prioritizes both maternal and fetal well-being.

Our hospital admitted a parturient with HTG-AP in June. Through active treatment and meticulous care during the perioperative period, the patient successfully gave birth. The following is the report of the nursing experience.

## 2. Case report

This is a single-patient case report.

### 2.1. General information

The patient, a 32-year-old female, was admitted to the intensive care unit (ICU) from the obstetrics department due to “pregnancy complicated with HTG-AP” on June 8, 2025. At the time of admission, the patient was conscious and able to move his limbs freely. The chief complaint was abdominal pain. The electrocardiogram monitoring indicated that the patient’s heart rate was 120 beats/min, the blood pressure 95/59 mm Hg, the respiratory rate 25 beats/min, and the pulse oximetry oxygen saturation (SpO_2_) 99%. The physical examination revealed that the patient’s bilateral pupils were equal in size and round, with a diameter of 2.5 mm, and the light reflex was present. The heart rhythm was regular, the heart sounds were slightly low, and no murmur was heard. The breath sounds in both lungs were clear, symmetrical on both sides, and no dry or wet rales were heard. The abdomen was soft and distended, consistent with the gestational age, no liver or spleen could be felt below the ribs, no tenderness or rebound pain was present, and there was no edema in both lower extremities. The blood gas analysis showed that PH was 7.409, PCO_2_ 23.9 mm Hg, PO_2_ 114 mm Hg, BE 9.9 mmol/L, blood glucose 6.86 mmol/L, lactate 1.58 mmol/L, hematocrit 35%, potassium 3.72 mmol/L, ionized calcium 0.86 mmol/L, and sodium 134 mmol/L.The patient had suffered from fatty liver for over 10 years without proper treatment, and underwent a lower segment cesarean section in 2012. She had a history of blood transfusion and had undergone plasma exchange twice 1 month ago. There was no history of trauma or drug allergy.

### 2.2. Diagnostic reasoning

(1) Acute cholecystic colic: This diagnosis is currently excluded based on an unremarkable urinalysis, negative Murphy’s sign, and a normal gallbladder ultrasound.
(2) Acute appendicitis: The absence of tenderness at McBurney’s point, along with an ultrasound finding of left renal effusion, made this diagnosis unlikely.
(3) Nephropyelitis: The presence of intermittent colicky abdominal pain and left renal effusion on ultrasound warrants that this diagnosis remains a consideration.
(4) Torsion of the pedicle of ovarian cyst: Neither the patient’s history nor the prenatal ultrasound reported ovarian cysts, which does not support the diagnosis.
(5) The patient’s acute presentation of persistent upper abdominal pain, accompanied by a serum lipase level 3 times the upper limit of normal and severely elevated triglycerides (>11.3 mmol/L), led to the diagnosis of HTG-AP.

### 2.3. Diagnostic challenges

The treatment of HTG-AP in pregnancy involves navigating conflicts between maternal-fetal safety and standard therapies. Key challenges include the bleeding risk from anticoagulation, drugs for maintaining pregnancy and promoting fetal lung maturity conflicting with the treatment of HTG-AP, and the need for fetal-safe antibiotics.

### 2.4. Treatment and outcome

After admission, the patient’s triglyceride levels continued to rise. Due to the contraindications of using drugs to lower triglycerides during pregnancy and the ineffectiveness of such measures,^[8]^ on the second day of admission, after consultation and discussion, a plasma exchange procedure was performed. The exchange fluid consisted of 2000 mL of homologous fresh frozen plasma combined with 20 g of 20% human albumin. The plasma exchange rate was 25 mL/min, and the whole blood exchange rate was 120 mL/min. Citrate sodium was used for anticoagulation. The treatment duration was 2 hours. The laboratory department returned a critical value for blood calcium on the same day, which was 1.30 mmol/L. Due to the fact that the drugs used for fetal protection and promoting fetal lung maturation were incompatible with the treatment for acute pancreatitis,^[9]^ the doctor’s orders were followed, and magnesium sulfate and dexamethasone were suspended, and 20 mL of 10% gluconate calcium was intravenously injected slowly. On the fifth day of admission, the patient’s blood lipid levels slightly decreased compared to before, bilirubin levels increased, and an MRI of the upper abdomen indicated severe pancreatic exudation involving the lungs and kidneys, with a risk of progressing to severe pancreatitis. The patient was 30 weeks and 1 day pregnant. The fetal ultrasound indicated that the fetus was normal. The current drug treatment options were limited. After the obstetrician assessed the risks and communicated the condition with the family, an emergency cesarean section was performed. The patient underwent a lower segment cesarean section under spinal anesthesia, using the LSA method to assist in the delivery of a live baby girl. The newborn, due to premature birth, scored 7 points on the 1-minute Apgar score, and after 5 minutes of rescue, the score was 8 points. The newborn was transferred to the pediatric department for further treatment.

When the patient was transferred from the operating room to the ICU, she was basically conscious, responded to calls by opening her eyes, and could answer simple questions. Both of his limbs moved in accordance with instructions. She complained of pain at the incision site, which was tolerable. There were no other special discomforts. Nasal catheter oxygen was administered. The electrocardiogram monitoring showed that heart rate was 100 beats/min, blood pressure 123/73 mm Hg, respiratory rate 19 beats/min, SpO_2_ 93%. The physical examination revealed that the bilateral pupils were equal in size and round, with normal light reflex. The heart rhythm was regular, no murmur was heard. The breath sounds in both lungs were clear and symmetrical. The abdomen was soft, and bowel sounds could be heard. The dressing at the incision site was dry, the drainage tube was unobstructed, and there was no obvious drainage fluid. After the operation, the patient was given fasting, gastrointestinal decompression, cefoperazone sulbactam sodium 4.5 g for anti-infection, pantoprazole sodium 40 mg for acid suppression, somatostatin to inhibit pancreatic fluid secretion, bezafibrate for lowering blood lipids, oxytocin to promote uterine contraction, rhubarb for laxative, and mirabilite for external application to promote wound healing. At the same time, the patient’s water, electrolyte, and acid-base balance were maintained. After the operation, the VTE risk assessment was performed, and the postpartum assessment score was 2 points, indicating a high risk of VTE, with a bleeding risk, and there were no contraindications for mechanical prevention. Therefore, basic VTE prevention was given.

On the first day after cesarean section, the patient’s bilateral lower lung breath sounds were low, with the left lower lung being particularly pronounced. The SpO_2_ dropped to 85%. The patient was then switched to mask oxygen inhalation. After encouraging the patient to inflate a balloon to exercise lung function, the SpO_2_ rose to 95%, and the patient was switched to nasal cannula oxygen inhalation. The patient was not suitable for breastfeeding. After communicating with the family members, breastfeeding was discontinued. The patient’s inflammatory indicators were elevated, and levofloxacin was used in combination for anti-infection treatment. On the second day after the operation, the patient did not need to undergo blood purification temporarily, so the femoral vein dialysis tube was removed. The patient did not defecate yesterday, the glycerin suppository and lactulose were given to facilitate defecation. On the third day after the operation, the patient consumed a small amount of thin rice soup and experienced no gastrointestinal reactions. On the fourth day after the operation, the patient did not require respiratory or circulatory support treatment. The blood lipid levels had decreased compared to before, and the blood amylase and ascites amylase were normal. The patient continued to follow a low-fat liquid diet. After assessment by the obstetrician, the patient was transferred back to the obstetrics department for further treatment. The timeline is shown in Table S1, Supplemental Digital Content, https://links.lww.com/MD/R107.

## 3. Nursing care

### 3.1. Taking maternal and infant safety as the core, synchronously monitoring fetal distress and uterine contractions

The patient had pregnancy complicated with HTG-AP and a history of cesarean section, which increased the risk of this pregnancy and belonged to the extremely high-risk pregnancy group. The risk characteristics mainly manifested as the pathological and physiological changes of HTG-AP would significantly increase the risk of multiple organ failure.^[10]^ Secondly, the uterine scar caused by the cesarean section posed a potential risk of uterine rupture during the late pregnancy and the delivery process. During the period of pregnancy and childbirth, due to the increased tension of the uterus, silent uterine rupture may occur in the patient. Because of its concealed symptoms and unobvious progression, silent uterine rupture is prone to be missed in diagnosis, often leading to disastrous consequences such as fetal death and severe maternal bleeding, which endangers the lives of both the mother and the fetus.^[11]^ Therefore, our nursing focus is as follows: continuously perform electronic fetal heart rate monitoring and uterine contraction monitoring, being vigilant for silent uterine rupture and type III fetal heart patterns. Instruct the patient to count fetal movements every 2 hours and notify the responsible nurse immediately if any abnormalities are detected. Dynamically monitor uterine contraction intensity and scar tenderness to identify abnormal uterine contraction patterns. Test triglycerides, lipase and coagulation function every 4 to 6 hours.

### 3.2. Implement multi-disciplinary collaborative perioperative management to balance the timing of cesarean section and the treatment of pancreatitis

HTG-AP, as a rare but extremely dangerous acute and critical illness during pregnancy, has its own unique complexity and challenges in clinical management. Due to the special physiological changes during pregnancy and the pathological mechanism of HTG-AP, the patients often experience rapid disease progression and a high incidence of complications, which seriously threaten the safety of both the mother and the fetus.^[12]^ Therefore, a multidisciplinary collaboration team composed of departments such as obstetrics, ICU, gastroenterology, endocrinology, anesthesiology, operating room and neonatology should be established. Through regular joint consultations, individualized treatment plans can be formulated, and dynamic monitoring and evaluation can be implemented to achieve precise control and timely intervention of the patient’s condition.

Balancing the timing of cesarean section and the treatment of pancreatitis is the core challenge in the management of pregnancy complicated by HTG-AP. It requires precise adherence to the “two priorities” principle: when obstetric emergencies such as fetal distress or signs of uterine rupture occur, immediate cesarean section should be performed to save the fetus; if the main symptom is pancreatitis and the fetal condition is stable, then priority should be given to controlling the progression of pancreatitis and striving to extend the gestational age. At 30 weeks and 1 day of pregnancy, an upper abdominal MRI revealed severe pancreatic exudation, which affected the lungs and kidneys, and there was a risk of the condition worsening to severe pancreatitis. Therefore, an emergency cesarean section was performed. The entire decision-making process was evaluated in real time by a multidisciplinary team to ensure that the cesarean section was carried out within the optimal time window, avoiding the aggravation of pancreatitis due to premature surgery and not delaying the timing of obstetric intervention.

### 3.3. Dynamic monitoring of blood lipids and pancreatic function, and precise regulation of plasma exchange and insulin therapy

For patients with pregnancy complicated by HTG-AP, it is crucial to establish a strict dynamic monitoring system for blood lipids and pancreatic function, and to precisely regulate plasma exchange and insulin therapy.^[13–15]^ After admission, the patients actively completed relevant examinations. Laboratory indicators showed that the total cholesterol was 17.44 mmol/L, triglyceride was 20.25 mmol/L, apolipoprotein A was 1.85 g/L, apolipoprotein B was 1.75 g/L, amylase was 208.2 U/L, and lipase was 343.60 U/L. The patient had indications for plasma exchange, so a plasma exchange procedure was performed. The precautions were as follows: Continuous electrocardiogram monitoring is required, and blood pressure should be recorded every 30 minutes to be vigilant for the occurrence of hypotension. Implement hourly dynamic blood gas monitoring, with a focus on monitoring the ionized calcium level. By prophylactically infusing 20 mL of 10% gluconate calcium to antagonize the calcium ion chelation effect related to citrate anticoagulation, maintain Ca²⁺ > 1.0 mmol/L. After admission to the department, the patient underwent VTE risk assessment. The prenatal assessment form scored 4 points, indicating a high risk of VTE, with a bleeding risk, and there were no contraindications for mechanical prevention. Therefore, VTE basic and drug prevention were implemented, and lower extremity vascular ultrasound was completed. Insulin was intravenously infused at a rate of 0.1 to 0.3 U/kg/h. Serum TG levels were tested once every 12 to 24 hours. Insulin was discontinued when serum TG levels were ≤5.65 mmol/L. Strict monitoring of blood glucose was required, with random blood glucose maintained within the range of 6.1 to 8.3 mmol/L. Low molecular weight heparin was injected subcutaneously at a dose of 100 U/kg, with an interval of ≥12 hours. Alert for bleeding and monitor blood routine and DIC.

### 3.4. Precise airway management and pulmonary rehabilitation training to prevent postoperative ARDS and atelectasis

For the prevention of postoperative respiratory complications in patients with pregnancy complicated by HTG-AP, a systematic airway management strategy and a stepwise pulmonary rehabilitation training program should be implemented. Through multi-modal intervention, the incidence of acute respiratory distress syndrome and atelectasis can be effectively reduced, and the prognosis of patients can be improved. Specific measures were as follows: maintain a semi-recumbent position at an angle of >30° to reduce the pressure of the abdominal cavity on the diaphragm and improve ventilation efficiency. Assist patients to turn over axially every 2 hours to prevent pressure-related pneumonia. Routine nasal catheter oxygen supply, with the oxygen flow controlled at 2 to 4L/min, maintaining SpO₂ > 95%. Perform 3 times a day of pursed-lip breathing, each time for 5 minutes, with an inhalation-to-exhalation ratio of 1:2. Abdominal breathing exercises are conducted with the assistance of nurses, with hands placed on the abdomen to feel the breathing movement. 6 to 12 hours after surgery, guide patients to perform balloon blowing training, once every 2 hours, 6 to 8 times a day, with each training session lasting 3 to 5 minutes. The principle of progressive adjustment is to decrease the frequency, increase the duration, and enhance the intensity. If conditions permit, encourage patients to transition from sitting beside the bed to getting out of bed for activities.

### 3.5. Comprehensive psychological support and family empowerment to alleviate maternal negative emotions and guide long-term health management

Throughout the entire treatment process, the patient encountered multiple psychological difficulties and challenges. During the acute phase, the patient exhibited excessive concern about the fetal health, manifesting as persistent anxiety about the pregnancy outcome and doubts about the safety of the treatment. After entering the recovery period, the patient faced body image disorders and conflicts regarding the motherhood role. Especially, the difficulty in breastfeeding caused by treatment restrictions often led to self-denial of being an “incompetent mother.” In long-term health management, the patient experienced metabolic management burnout, felt hopeless about lifelong lipid control, and got trapped in a dual predicament of recurrence fear and genetic concerns when making decisions about future pregnancies. The difficulty in reconfiguring social relationships manifested as workplace discrimination and social avoidance.

The specific measures were as follows: the responsible nurse conducted structured stress reduction conversation intervention at 09:00 and 15:00 every day. In the first stage, open listening was implemented to establish a trust relationship with the patient by using techniques such as nodding and repetition; in the second stage, cognitive restructuring was carried out to guide the patient to identify 1 positive treatment progress; in the third stage, SMART principles were used to set small treatment goals for the day. A structured family meeting was held every day, involving the attending physician, the responsible nurse, and a representative of 1 family member. The responsible nurse displayed the trend chart of key indicators for the day on the electronic shared screen, and the attending physician interpreted the core indicators. The family member could ask questions based on the “treatment risk/fetal impact/prognosis judgment” template, and the doctor answered. Health management education was carried out during the rehabilitation period, including: customizing a low-fat diet plan, setting a progressive exercise plan, establishing a long-term follow-up mechanism, and conducting lipid and pancreatic function rechecks at 1, 3, and 6 months postpartum. Special attention was paid to the prevention of postpartum depression, providing a 6-month psychological counseling follow-up service, and guiding scientific contraception, comprehensively ensuring the long-term health of both mother and baby.

## 4. Limitations

This study has the following limitations: Firstly, as a single-center case report, the sample size is limited, and the generalizability of the conclusion needs to be verified through larger-sample multi-center studies; Secondly, the patient’s condition is complex and there are individual differences (such as previous cesarean section history, multiple plasma exchange history, etc), and the effect of nursing measures may be affected by confounding factors; Thirdly, retrospective studies may have biases, the complexity of multidisciplinary team collaboration, and the subjectivity of nursing intervention may affect the wide applicability and objectivity of the research results. To address these challenges, future research can expand the sample size, conduct prospective cohort studies, standardize nursing intervention, optimize data management, and conduct long-term follow-up to further improve the scientific and practicality of the research, thereby establishing evidence-based nursing norms for gestational HTG-AP.

## 5. Conclusion

This article reported the perioperative nursing experience of a patient with pregnancy complicated with HTG-AP undergoing cesarean section. Through multidisciplinary collaboration, an individualized plan was formulated to control the progression of pancreatitis and perform a cesarean section at an appropriate time. Precise nursing measures were also adopted, including dynamic lipid monitoring, plasma exchange management, postoperative respiratory support, and psychological intervention. Eventually, the patient successfully gave birth and her condition stabilized. This demonstrates the crucial role of multidisciplinary joint care in the treatment of pregnancy complicated with HTG-AP, providing a reference for the nursing practice of similar cases. Further exploration is needed to establish a standardized nursing pathway for HTG-AP during pregnancy and strategies to improve long-term maternal and infant outcomes.

## Acknowledgments

The authors thank the patient for providing consent to publish this case report.

## Author contributions

**Data curation:** Yinhao Yang, Juanjuan Lin, Bolun Zhang, Limin Cui.

**Methodology:** Yinhao Yang, Juanjuan Lin.

**Supervision:** Zhenzhen Yuan.

**Writing – original draft:** Yinhao Yang, Juanjuan Lin, Bolun Zhang, Limin Cui.

**Writing – review & editing:** Yinhao Yang, Juanjuan Lin, Zhenzhen Yuan.

## Supplementary Material


