# Supplementary material for: Perioperative nursing care for a parturient with hypertriglyceridemic acute pancreatitis undergoing cesarean section: A case report
Source: Medicine (Baltimore). 2026 Jan 9;105(2):e46944. doi: 10.1097/MD.0000000000046944 (PMC12794990; doi:10.1097/MD.0000000000046944)
Supplement: Supplementary file 1 [file medi-105-e46944-s001.pdf]

Table 1 Timeline Table

Clinical events	Timeline	Major interventions
Obstetrics Admission	June 8, 2025, at 2:31 PM	Initiated Medical Management: MDT, fluid resuscitation, NPO status.<br>Procedures & Monitoring: GI decompression, continuous ECG monitoring, oxygen therapy.<br>Status & Documentation: Critical condition communicated to family member. 24-hour I/O closely monitored.
ICU Transfer	June 8, 2025, at 8:45 PM	Transferred to ICU from Obstetrics following resuscitation due to unstable vital signs.
Critical Care	June 8 to 11, 2025	Medication: MgSO□ 4g IV pump (Jun 8 to 9), Insulin 0.1-0.3 U/kg/h IV pump (Jun 8 to 9), Dexmedetomidine 0.2mg IV pump (Jun 10 to 11), Pantoprazole 40mg IV bid (Jun 9 to 11), Ceftazidime 2g IV tid (Jun 9 to 11), Human Albumin 20g IV & Furosemide 20mg IVP qd (Jun 10 to 11), Enoxaparin 4000 IU SC qd (Jun 9 to 11).<br>Therapy: Plasma Exchange (01:00 - 03:00, Jun 9).
Caesarean Section	June 12, 2025	Mother: Delivered a live female infant via LSCS under spinal anesthesia.<br>Newborn: Preterm, Apgars 7 (1-min) & 8 (5-min).<br>Action: Newborn transferred to Pediatrics for further care.
ICU Transfer	June 12, 2025, at 1:55 PM	Medication: Hydromorphone 6mg IV pump (Jun 12 to 16), Somatostatin 6mg IV pump (Jun 12 to 16), Magnesium Sulfate 1g IV once, Dexamethasone Sodium Phosphate 5mg IV once, Oxytocin 10 IU IV qd (Jun 12 to 15), Cefoperazone-Sulbactam 3g IV tid (Jun 12 to 15), Pantoprazole 40mg IV bid (Jun 12 to 15), Calcium Gluconate 2g IV bid, PRBC 1.5 units IV qd, Budesonide 1mg & Ipratropium 0.5mg neb tid (Jun 12 to 16), Bezafibrate 0.4g NG qd (Jun 12 to 16).
POD #1	June 13, 2025	Desaturation (85%): Mask O2 → nasal cannula, incentive spirometry (O2 sat ↑ to 95%).<br>Lactation suppression: Vitamin B6 200mg & Huiru Bao 50ml NG tid (Jun 13 to 15).<br>Elevated inflammatory markers: Levofloxacin 0.5g IV qd (Jun 13 to 15) was added.<br>Atelectasis/ARDS (per MRI & lung exam): Continued pulmonary toileting, Ambroxol 15mg IVP tid (Jun 13 to 15).<br>VTE Prophylaxis: Switched to Nadroparin 3000 IU SC qd (Jun 13 to 15).<br>For constipation: Rhubarb 3g NG tid (Jun 13 to 16).

Continued Table1 Timeline Table

POD #2	June 14, 2025	Pulmonary: Continued incentive spirometry. CXR shows left lower lung infection. Oxygenation slightly improved.<br>Abdominal: Ascites decreased per US. Paracentesis sent for labs. Lactulose 10ml NG tid added for constipation management.<br>Treatment: Hemodialysis catheter removed.
POD #3	June 15, 2025	Lungs: Respiratory status improved.Continued pulmonary toileting (incentive spirometry).<br>Abdomen: Bowel sounds returned. Plan to start clear liquid diet.
POD #4	June 16, 2025	Status: No respiratory/circulatory support needed.<br>Diet: Continue low-fat liquid diet.<br>Plan: Cleared for transfer back to obstetrics.
Obstetrics Transfer	June 16, 2025, at 11:51 AM	VTE Prophylaxis: High-risk score. Initiated LMWH 5000IU SC & pneumatic compression.<br>Activity/Diet: Low-fat liquid diet. Foley removed for ambul[illegible]<br>Meds: Continued dual antibiotics, somatostatin, bezafibrate.<br>Plan: GI consult. Pulmonary toileting. Monitor labs & I/O.
